# Assessment of Hospital Readmissions From the Emergency Department After Implementation of Medicare’s Hospital Readmissions Reduction Program

**DOI:** 10.1001/jamanetworkopen.2020.3857

**Published:** 2020-05-01

**Authors:** Charleen Hsuan, Brendan G. Carr, Renee Y. Hsia, Geoffrey J. Hoffman

**Affiliations:** 1 Department of Health Policy and Administration, Pennsylvania State University, University Park; 2 Department of Emergency Medicine, Icahn School of Medicine at Mount Sinai, New York, New York; 3 Department of Emergency Medicine, University of California, San Francisco; 4 Department of Systems, Populations and Leadership, University of Michigan School of Nursing, Ann Arbor

## Abstract

**Importance:**

The Medicare Hospital Readmissions Reduction Program (HRRP) is associated with reduced readmission rates, but it is unknown how this decrease occurred.

**Objective:**

To examine whether the HRRP was associated with changes in the probability of readmission at emergency department (ED) visits after hospital discharge (ED revisits) overall and depending on whether admission is typically indicated for the patient’s condition at the ED revisit.

**Design, Setting, and Participants:**

This retrospective cohort study used hospital and ED discharge data from California, Florida, and New York from January 1, 2010, to December 31, 2014. A difference-in-differences analysis examined change in readmission probability at ED revisits for recently discharged patients; ED revisits with clinical presentations for which admission is typically indicated vs those for which admission is more variable (ie, discretionary) were examined separately. Inclusion criteria were Medicare patients 65 years and older who revisited an ED within 30 days of inpatient discharge. Data were analyzed from December 18, 2018, to September 11, 2019.

**Exposures:**

Before and after HRRP implementation among patients initially hospitalized for targeted vs nontargeted conditions.

**Main Outcomes and Measures:**

Thirty-day unplanned hospital readmissions at the ED revisit.

**Results:**

A total of 9 914 068 index hospitalizations were identified in California, Florida, and New York from 2010 to 2014. Of 2 052 096 discharges in 2010, 1 168 126 (56.9%) discharges were women and 566 957 discharges (27.6%) were among patients older than 85 years. Among 1 421 407 patients with an unplanned readmission within 30 days of discharge, 1 266 107 patients (89.1%) were admitted through the ED. A total of 1 906 498 ED revisits were identified. After adjusting for patient demographic and clinical characteristics from the index hospitalization, HRRP implementation was associated with fewer readmissions from the ED, with a difference-in-difference estimate of −0.9 (95% CI, −1.4 to −0.4) percentage points (*P* < .001), or a 1.4% relative decrease from the 65.8% pre-HRRP readmission rates. Implementation of the HRRP was associated with fewer readmissions at the ED revisit involving clinical presentations for which admission is typically indicated (difference-in-differences estimate, −1.1 [95% CI, −1.6 to −0.6] percentage points; *P* < .001), or a 1.2% relative decrease from the 93.6% pre-HRRP rate. These results appear to be associated with patients presenting at the ED revisit with congestive heart failure (difference-in-difference estimate, −1.2 [95% CI, −2.0 to −0.4] percentage points; *P* = .003).

**Conclusions and Relevance:**

These findings suggest that implementation of the HRRP was associated with a lower likelihood of readmission for recently discharged patients presenting to the ED, specifically for congestive heart failure. This highlights the critical role of the ED in readmission reduction under the HRRP and suggests that patient outcomes after HRRP implementation should be further studied.

## Key Points

**Question:**

Is Medicare’s Hospital Readmissions Reduction Program associated with changes in the probability of readmission for discharged patients revisiting the emergency department (ED)?

**Findings:**

In this cohort study including data for 9 914 068 patients, the Hospital Readmissions Reduction Program was associated with a 1.4% decrease in readmissions at the ED revisit. The program was associated with 1.2% fewer readmissions from the ED involving clinical presentations for which admission is usually indicated.

**Meaning:**

These findings suggest that the Hospital Readmissions Reduction Program was associated with changes in the probability of readmission for certain conditions, highlighting the critical and evolving role of the ED in hospital readmission patterns.

## Teaser

This cohort study examines the association of the implementation of Medicare’s Hospital Readmissions Reduction Program (HRRP) with changes in the probability of readmission among recently discharged patients revisiting the emergency department.

## Introduction

Medicare’s Hospital Readmissions Reduction Program (HRRP) penalized hospitals more than $500 million for excess readmissions rates in 2017,^1^ providing incentives for hospitals to decrease readmissions.^2^ Although the program is associated with lower readmission rates,^3^ it is unclear how this decrease occurred. While some of the change may be associated with improved posthospitalization care coordination and outpatient care provided outside of the hospital setting,^4,5,6,7,8^ another possibility might be changes in ED disposition, as hospitals can avoid readmissions by increasing their threshold to readmit recently discharged patients who visit the ED (ie, ED revisit).^9^

While researchers have examined associations of the HRRP, implemented in 2012, with changes in postdischarge care quality and penalty avoidance, it is less clear whether the HRRP is associated with a change in the likelihood of readmission at an ED revisit. We sought to determine how readmission patterns from the ED changed after implementation of the HRRP.

Previous studies exploring the HRRP and ED use have examined changes in ED use^10^ or observation stays.^3^ A 2018 Medicare Payment Advisory Commission report^11^ found that the HRRP was not associated with a change in overall ED visits, and other studies have reported that the HRRP was associated with no or slightly increased treat-and-release ED revisits.^12,13,14^ These findings suggest that when recently discharged patients visit the ED, the likelihood of being readmitted from the ED may have decreased. However, to our knowledge, this possibility has not been examined directly. Furthermore, we are unaware of studies examining whether changes in readmission risk in the ED are associated with care patterns inconsistent with typical care. To address this gap, we conducted a difference-in-difference analysis to estimate whether the HRRP was associated with a change in readmission risk at an ED revisit and whether the change in risk varied for presentations at the ED visit for conditions for which a hospital admission is variable (eg, urinary tract infection) vs typically indicated (eg, septicemia).^15^ Our findings may help clarify the role of the ED in hospital readmissions and provide insight into how the HRRP may have influenced hospital behavior.

## Methods

This study was approved by the Pennsylvania State University institutional review board, which waived informed consent because this was a secondary analysis of preexisting data. This study is reported following the Strengthening the Reporting of Observational Studies in Epidemiology (STROBE) reporting guideline.

### Data Sources and Setting

We merged hospital and ED data from California, Florida, and New York with data on hospital characteristics from the American Hospital Association’s Annual Survey. Because of the shift from *International Classification of Diseases, Ninth Revision*^16^ coding to *Tenth Revision*^17^ coding in 2015, all data were from January 1, 2010, to December 31, 2014. Data were obtained through California’s Office of Statewide Health and Planning (OSHPD), and through the State Emergency Department Databases and the State Inpatient Databases from the Healthcare Cost and Utilization Project (HCUP). California, Florida, and New York are 3 of the 4 states with the greatest number of Medicare patients; these 3 states comprise approximately one-fourth of the country’s Medicare population^18^ and represent approximately one-fifth of participating hospitals.

We followed Centers for Medicare & Medicaid Services (CMS) methods by beginning with identifying eligible index discharges from short-term acute hospitals and excluding hospitals not participating in the HRRP (eg, critical access hospitals) for all Medicare beneficiaries 65 years and older.^19^ Patients with index hospitalizations for conditions later targeted by the HRRP^20^ were excluded in the main analysis to account for hospitals anticipating changes to the policy, but these patients were included in sensitivity analyses.

An ED revisit was defined as the first ED visit that occurred within 30 days of discharge from an index hospitalization, regardless of whether the patient was admitted (ie, treat-and-release ED revisits and readmissions at the ED revisit). Following CMS guidelines, index discharges involving inpatient death or transfers were excluded; ED revisits were excluded if the ED revisit involved death, transfer, or planned procedures or diagnoses (eg, chemotherapy).^19^ We also excluded 0.002% of discharges in rural hospitals (eFigure 1 in the Supplement).

### Participants and Variables

Our main outcome was whether the patient was readmitted at the ED revisit. The main variable was a difference-in-difference variable indicating whether the discharged patient visited the ED before or after HRRP implementation and whether the patient was initially hospitalized for a condition that was targeted under the HRRP (ie, conditions for which hospitals could be penalized) vs one that was nontargeted. The targeted conditions were acute myocardial infarction (AMI), heart failure, and pneumonia.^19^ Following previous research,^3,21,22^ nontargeted conditions were all conditions not specifically targeted by the HRRP.

To assess whether readmissions from EDs varied by the type of condition at the ED revisit, we identified ED revisit conditions for which admission is potentially nondiscretionary compared with those with more variable admission rates. To do so, we relied on the 2015 study by Venkatesh et al,^15^ which examined clinical conditions frequently admitted from the ED and classified conditions by whether patients presenting in the ED with these conditions were almost always admitted (hereafter, *potentially nondiscretionary admission*) compared with conditions for which there was high variability in whether patients were admitted (hereafter, *discretionary admission*). The conditions for which admission is potentially nondiscretionary were septicemia, AMI, acute cerebrovascular disease, and congestive heart failure (CHF). The conditions for which admission is discretionary were mood disorders, nonspecific chest pain, skin and soft tissue infections, urinary tract infections, and chronic obstructive pulmonary disease.

### Statistical Analysis

We compared discharge, patient, and hospital characteristics for targeted and nontargeted conditions using χ^2^ and *t* tests for categorical and continuous variables. A 2-sided *P* < .05 was considered statistically significant for all hypothesis tests. Data were analyzed from December 18, 2018, to September 11, 2019.

We performed difference-in-differences analyses at the patient discharge level (eAppendix 1 and eFigure 1 in the Supplement). We estimated the probability of readmission at the ED revisit as a function of time-varying beneficiary and hospital characteristics, whether the index hospitalization involved a targeted or nontargeted diagnosis, an indicator for the HRRP implementation period (ie, October 2012), and an interaction between the targeted condition indicator and the HRRP year indicator (the difference-in-differences estimate) (eAppendix 2 in the Supplement). A negative difference-in-differences coefficient indicates that the HRRP is associated with a lower likelihood of readmission at the ED revisit.

Patient characteristics were age (categorized in 5-year bands), sex, race/ethnicity (classified by HCUP and OSHPD), Charlson Comorbidity Index score at the index hospitalization,^23^ whether the ED revisit was on a weekend, and the service line at the index hospitalization, as definied by CMS.^19^ We included race/ethnicity in the risk adjustment as a proxy for socioeconomic factors associated with readmission risk.^24^ To account for changes in the number of reportable diagnosis codes during the study period that may influence results,^21^ we used only the first 9 secondary diagnoses in risk adjustment. Hospital characteristics were size, teaching status, metropolitan or urban designation, and ownership (eAppendix 2 in the Supplement). These patient and hospital characteristics adjusted for ED revisit and readmission risks.^25,26,27,28^ We accounted for secular trends and area-level factors that may be associated with readmission risk by including year and state dummy variables. To adjust for clustering of patients within hospitals, we used cluster-robust SEs.

We also stratified by the condition at the ED revisit: ED presentations for which admission is potentially nondiscretionary vs discretionary. We further divided conditions for which admission is potentially nondiscretionary into CHF and all other, as a study by Collins et al has suggested that admission for CHF is not always necessary.^29^ We specified linear probability models, which are frequently used to estimate the probability of readmissions in the context of national policy changes,^30^ including the HRRP.^13^

In robustness checks, we examined alternative specifications (accounting for monthly trends, logistic regression, and hospital fixed-effects to control for unobservable hospital factors), modified the outcome to account for potential differences in care patterns (transfers and observation stays), included all secondary diagnoses in risk adjustment, and controlled for community-level factors associated with postacute care that could confound the observed relationship (eAppendix 3 in the Supplement).^24,31,32^ We conducted a falsification test in which we examined readmissions for patients revisiting the ED 31 to 45 days after index discharge. Since the HRRP would not penalize these readmissions, we did not expect to see differences in readmission at the ED revisit that occur then. We also examined the probability of an ED revisit to aid in the interpretation of our results (eTable 2 in the Supplement).

## Results

We identified 9 914 068 index hospitalizations in California, Florida, and New York from 2010 to 2014. Of 2 052 096 discharges in 2010, 1 168 126 (56.9%) of these discharges were women; 331 331 (16.2%) of visits were from patients aged 65 to 69 years; 357 895 (17.4%) from patients aged 70 to 74 years; 385 914 (18.8%) from patients aged 75 to 79 years; 409 999 (20.0%) from patients aged 80 to 84 years; and the remainder from patients 85 years or older (566 957 patients [27.6%]). Within 30 days of discharge, patients with targeted conditions visited the ED more often than patients with nontargeted conditions (283 624 patients [26.4%] vs 1 773 286 patients [21.7%]; *P* < .001) and experienced a readmission more often (223 768 patients [20.6%] vs 1 318 543 patients [15.9%]; *P* < .001) (eTable 1 in the Supplement). Among 1 421 407 patients with an unplanned admission, 1 266 107 patients (89.1%) were admitted through the ED, although patients with targeted conditions1 were more likely to be admitted through the ED than patients with nontargeted conditions (192 988 patients [91.9%] vs 1 073 119 patients [88.6%]; *P* < .001).

A total of 1 906 498 ED revisits occurred within 30 days of an index hospitalization. Of those, most patients (1 649 509 patients [86.5%]) had conditions at the index hospitalization that were not targeted by the HRRP. Patients with targeted conditions more often presented at the ED revisit with conditions for which admission is potentially nondiscretionary than did patients with nontargeted conditions (68 142 patients [26.5%] vs 212 433 patients [12.9%]; *P* < .001). Prior to HRRP implementation, patients with index hospitalizations targeted under the HRRP differed significantly from those not targeted under the HRRP for almost every characteristic (Table 1): they were more likely to be older (age ≥85 years: 51 287 patients [33.5%] vs 272 913 patients [28.8%] *P* < .001) and have fewer comorbidities (0-1 comorbidity: 70 510 patients [46.1%] vs 584 506 patients [61.6%]; *P* < .001), and they were less likely to be white (102 645 patients [67.1%] vs 639 496 patients [67.4%]; *P* < .001) and women (78 711 patients [51.5%] vs 539 631 patients [56.9%]; *P* < .001). Patients with targeted conditions were more likely than patients with nontargeted conditions to be treated in smaller hospitals (quintile 1 hospitals: 8714 patients [5.7%] vs 46 325 patients [4.9%]; *P* < .001), public hospitals (19 645 patients [12.8%] vs 114 512 patients [12.1%]; *P* < .001) or for-profit hospitals (30 405 patients [19.9%] vs 180 354 patients [19.0%]; *P* < .001), and less likely to be treated in hospitals in metropolitan areas (145 392 patients [95.0%] vs 910 502 patients [96.0%]; *P* < .001).

**Table 1.** Descriptive Characteristics of Discharges and Patient and Hospital Characteristics Before Hospital Readmissions Reduction Program Implementation Stratified by Condition at ED Revisit

Condition	ED revisits, No. (%)^a^					
	Targeted condition admissions			Nontargeted condition admissions		
	All (n=152 983)	Discretionary (n=15 626)^b^	Potentially nondiscretionary (n=40 821)^c^	All (n=948 752)	Discretionary (n=98 214)^b^	Potentially nondiscretionary (n=118 496)^c^
**Patient characteristics**						
Age, y						
65-69	19 961 (13.1)	2389 (15.3)	4924 (12.1)	150 809 (15.9)	15 890 (16.2)	14 245 (12.0)
70-74	23 158 (15.1)	2626 (16.8)	5946 (14.6)	159 424 (16.8)	16 365 (16.7)	17 136 (14.5)
75-79	27 009 (17.7)	2857 (18.3)	7027 (17.2)	175 221 (18.5)	18 104 (18.4)	20 812 (17.6)
80-84	31 568 (20.6)	3191 (20.4)	8482 (20.8)	190 385 (20.1)	19 638 (20.0)	24 828 (21.0)
≥85	51 287 (33.5)	4563 (29.2)	14 442 (35.4)	272 913 (28.8)	28 217 (28.7)	41 475 (35.0)
Women	78 711 (51.5)	8578 (54.9)	20 512 (50.3)	539 631 (56.9)	60 070 (61.2)	65 631 (55.4)
Race/ethnicity						
White	102 645 (67.1)	11 126 (71.2)	26 090 (63.9)	639 496 (67.4)	68 342 (69.6)	78 948 (66.6)
Black	16 738 (10.9)	1460 (9.3)	5099 (12.5)	106 712 (11.3)	9953 (10.1)	13 805 (11.7)
Hispanic	22 760 (14.9)	2143 (13.7)	6567 (16.1)	134 626 (14.2)	13 933 (14.2)	16231 (13.7)
Asian or Pacific Islander	6075 (4.0)	464 (3.0)	1722 (4.2)	36 929 (3.9)	2989 (3.0)	5715 (4.8)
Other	4226 (2.8)	373 (2.4)	1239 (3.0)	27 573 (2.9)	2620 (2.7)	3458 (2.9)
Missing	539 (0.4)	60 (0.4)	104 (0.3)	3416 (0.4)	377 (0.4)	339 (0.3)
Charlson Comorbidity Index score						
0	26 469 (17.3)	2653 (17.0)	5610 (13.7)	359 668 (37.9)	36 758 (37.4)	32 683 (27.6)
1	44 041 (28.8)	5151 (33.0)	11 397 (27.9)	224 838 (23.7)	26 556 (27.0)	30 842 (26.0)
2	34 944 (22.8)	3622 (23.2)	9613 (23.6)	192 265 (20.3)	19 005 (19.4)	26 426 (22.3)
3	29 517 (19.3)	2517 (16.1)	9018 (22.1)	101 084 (10.7)	9641 (9.8)	17 675 (14.9)
4	12 392 (8.1)	1219 (7.8)	3900 (9.6)	34 747 (3.7)	3442 (3.5)	6655 (5.6)
≥5	5620 (3.7)	464 (3.0)	1283 (3.1)	36 150 (3.8)	2812 (2.9)	4215 (3.6)
ED visit on a weekend	40 348 (26.4)	4145 (26.5)	10 959 (26.9)	250 519 (26.4)	26 564 (27.1)	31 294 (26.4)
**Hospital characteristics**						
Size, annual admissions						
Quintile 1	8714 (5.7)	1022 (6.5)	1932 (4.7)	46 325 (4.9)	5174 (5.3)	5197 (4.4)
Quintile 2	16 876 (11.0)	1850 (11.8)	4244 (10.4)	97 175 (10.2)	10 284 (10.5)	12 149 (10.3)
Quintile 3	26 148 (17.1)	2769 (17.7)	6859 (16.8)	155 186 (16.4)	16 280 (16.6)	19 523 (16.5)
Quintile 4	41 733 (27.3)	4242 (27.2)	11 309 (27.7)	257 828 (27.2)	26 720 (27.2)	32 639 (27.5)
Quintile 5	59 512 (38.9)	5743 (36.8)	16 477 (40.4)	392 238 (41.3)	39 756 (40.5)	48 988 (41.3)
Academic	51 745 (33.8)	4834 (30.9)	14 501 (35.5)	350 226 (36.9)	34 779 (35.4)	43 939 (37.1)
Ownership						
Public	19 645 (12.8)	1996 (12.8)	5201 (12.7)	114 512 (12.1)	12 292 (12.5)	13 031 (11.0)
Nonprofit	102 933 (67.3)	10 440 (66.8)	27 079 (66.3)	653 886 (68.9)	66 408 (67.6)	82 506 (69.6)
For-profit	30 405 (19.9)	3190 (20.4)	8541 (20.9)	180 354 (19.0)	19 514 (19.9)	22 959 (19.4)
Metropolitan	145 392 (95.0)	14 723 (94.2)	38 943 (95.4)	910 502 (96.0)	93 947 (95.7)	114 026 (96.2)

Abbreviation: ED, emergency department.

^a^ An ED revisit is defined as an ED visit occurring within 30 days of the date of discharge from an eligible index hospitalization.

^b^ Includes mood disorders, nonspecific chest pain, skin and soft tissue infections, urinary tract infections, and chronic obstructive pulmonary disease.

^c^ Includes septicemia, acute myocardial infarction, acute cerebrovascular disease, and congestive heart failure.

In adjusted analyses, before HRRP implementation, 65.8% (95% CI, 64.9% to 66.6%) of patients with 30-day ED revisits after an index hospitalization for a targeted condition were readmitted at the ED revisit, compared with 64.8% (95% CI, 64.0% to 65.7%) of patients with ED revisits after implementation of the HRRP, a decrease in readmission probability of 1.0 (95% CI, −1.6% to −0.3%) percentage points (*P* = .005) (Table 2). There was no significant change in the likelihood of readmissions at the ED revisit for patients with index hospitalizations not targeted by the HRRP (before: 59.4% [95% CI, 58.9% to 60.2%]; after: 59.4% [95% CI, 58.6% to 60.2%]; *P* = .83). In all, the HRRP was associated with a decrease of 0.9 (95% CI, −1.4 to −0.4) percentage points in the likelihood of readmission at the ED revisit (*P* < .001), or a 1.4% decrease from the pre-HRRP readmission rates.

**Table 2.** Readmissions at Emergency Department Revisit

Condition	Estimated probability, % (95% CI)^a^		Post-HRRP to pre-HRRP implementation difference, percentage points (95% CI)	*P* value	Difference-in-difference, percentage points (95% CI)	*P* value
	Pre-HRRP implementation	Post-HRRP implementation				
Targeted	65.8 (64.9 to 66.6)	64.8 (64.0 to 65.7)	−1.0 (−1.6 to −0.3)	.005	−0.9 (−1.4 to −0.4)	<.001
Nontargeted	59.4 (58.9 to 60.2)	59.4 (58.6 to 60.2)	−0.1 (−0.6 to 0.5)	.83		

Abbreviation: HRRP, Hospital Readmissions Reduction Program.

^a^ Controlled for age, sex, race/ethnicity, Charlson Comorbidity Index score, whether the ED revisit was on a weekend, patient’s condition at the index hospitalization, hospital size, teaching status, metropolitan or urban designation, ownership status, state, and year.

In stratified analyses, 93.6% (95% CI, 93.0% to 94.1%) of patients with an index hospitalization for a targeted condition who presented at an ED revisit with conditions for which admission is potentially nondiscretionary were readmitted, a probability that decreased to 92.7% (95% CI, 92.1% to 93.3%) after HRRP implementation, a decrease of 0.9 (95% CI, −1.6 to −0.2) percentage points in readmission probability (*P* = .01) (Table 3). In comparison, 93.4% (95% CI, 93.0% to 93.9%) of patients with an index hospitalization for a nontargeted condition who presented at the ED revisit with these same 4 conditions were readmitted, a probability that did not change after HRRP implementation (difference, 0.2 [95% CI, −0.3 to 0.7] percentage points; *P* = .42). The difference-in-difference estimate suggested that the HRRP was associated with a decrease of 1.1 (95% CI, −1.6 to −0.6) percentage points in the likelihood of readmission at the ED revisit for patients presenting to the ED with conditions for which admission is potentially nondiscretionary (*P* < .001), or a 1.2% decrease from the pre-HRRP readmission rate. When further stratified by condition at the ED revisit, these results seem to be driven by patients presenting to the ED with CHF. Specifically, the HRRP was associated with a decrease of 1.2 (95% CI, −2.0 to −0.4) percentage points in the likelihood of readmission at the ED revisit for patients presenting at the ED with CHF (*P* = .003), but the decrease for patients presenting at the ED with septicemia, AMI, and acute cerebrovascular disease was not statistically significant. There was a decrease in readmissions at the ED revisit for conditions for which admission was discretionary, but the difference was not statistically significant (Table 4).

**Table 3.** Readmissions at the ED Revisit for Conditions for Which Admission Is Potentially Nondiscretionary^a^

Condition	Estimated probability, % (95% CI)^b^		Post-HRRP to pre-HRRP implementation difference, percentage points (95% CI)	*P* value	Difference-in-difference, percentage points (95% CI)	*P* value
	Pre-HRRP implementation	Post-HRRP implementation				
All						
Targeted	93.6 (93.0 to 94.1)	92.7 (92.1 to 93.3)	−0.9 (−1.6 to −0.2)	.01	−1.1 (−1.6 to −0.6)	<.001
Nontargeted	93.4 (93.0 to 93.9)	93.7 (93.2 to 94.1)	0.2 (−0.3 to 0.7)	.42		
Patients presenting in ED with CHF only						
Targeted	89.6 (88.9 to 90.4)	88.7 (87.8 to 89.6)	−1.0 (−2.1 to 0.1)	.09	−1.2 (−2.0 to −0.4)	.003
Nontargeted	88.5 (87.8 to 89.2)	88.7 (87.8 to 89.6)	0.2 (−0.8 to 1.3)	.66		
Patients presenting in ED with septicemia, AMI, or acute cerebrovascular disease						
Targeted	96.3 (95.8 to 96.8)	96.3 (95.7 to 96.9)	0 (−0.6 to 0.7)	.96	−0.1 (−0.7 to 0.4)	.61
Nontargeted	97.1 (96.9 to 97.4)	97.3 (97.0 to 97.6)	0.2 (−0.3 to 0.6)	.50		

Abbreviations: AMI, acute myocardial infarction; CHF, congestive heart failure; ED, emergency department; HRRP, Hospital Readmissions Reduction Program.

^a^ ED diagnoses where admission is potentially nondiscretionary are septicemia, AMI, acute cerebrovascular disease, and CHF.

^b^ Controlled for age, sex, race/ethnicity, Charlson Comorbidity Index score, whether the ED revisit was on a weekend, patient’s condition at the index hospitalization, hospital size, teaching status, metropolitan or urban designation, ownership status, state, and year.

**Table 4.** Readmissions at the ED Revisit for Conditions for Which Admission Is Discretionary^a^

Condition	Estimated probability, % (95% CI)^b^		Post-HRRP to pre-HRRP implementation difference, percentage points (95% CI)	*P* value	Difference-in-difference	*P* value
	Pre-HRRP implementation	Post-HRRP implementation				
Targeted	55.4 (53.8 to 57.0)	55.5 (53.6 to 57.4)	0.1 (−1.7 to 1.9)	.92	−0.9 (−2.1 to 0.4)	.18
Nontargeted	53.4 (52.1 to 54.7)	54.4 (53.0 to 55.8)	1.0 (−0.4 to 2.4)	.17		

Abbreviations: ED, emergency department; HRRP, Hospital Readmissions Reduction Program.

^a^ Includes mood disorders, nonspecific chest pain, skin and soft tissue infections, urinary tract infections, and chronic obstructive pulmonary disease.

^b^ Controlled for age, sex, race/ethnicity, Charlson Comorbidity Index score, whether the ED revisit was on a weekend, patient’s condition at the index hospitalization, hospital size, teaching status, metropolitan or urban designation, ownership status, state, and year.

The main results are robust, as seen in the robustness checks (eTables 3, 4, 5, and 6 in the Supplement). Notably, when we analyzed ED revisits among patients who had been discharged 31 to 45 days after the index discharge—when readmissions are no longer penalized under the HRRP—we no longer observed a statistically significant decrease in the probability of readmission.

## Discussion

The findings of this cohort study highlight the importance of the ED in Medicare’s HRRP.^33^ Our results suggest that implementation of the HRRP was associated with a 1.4% decrease in the likelihood of readmission for patients presenting to the ED within 30 days after an index discharge. These findings, together with our observation that almost 90% of unplanned readmissions originated in the ED, suggest that engaging the ED in alternative payment models, care coordination, and penalty avoidance is essential.^34^ A 2019 study by Ody et al^21^ indicated that the HRRP was associated with a 2.5% reduction in readmission rates for targeted vs nontargeted conditions. If our results generalize, we estimate that approximately half of this observed reduction in readmissions may be associated with the decrease in the likelihood of readmissions after ED visits.^21^ This reduction in readmissions from the ED was most pronounced in patients presenting to the ED with clinical conditions for which admission is potentially nondiscretionary (particularly CHF), with no change observed among patients presenting to the ED with conditions for which admission is discretionary.

The influence of the HRRP on ED disposition may reflect changes in the treatment of recently hospitalized patients consistent with CMS’s intent to improve discharge planning, patient education, and other initiatives to coordinate care and transition patients out of the hospital.^8,35,36^ The decrease in readmissions from the ED may suggest that the ED provides recently discharged patients a means of addressing exacerbations of illness while avoiding subsequent hospitalization, serving as a readmission safety net. If readmissions prior to HRRP implementation were a result of inadequate outpatient resources or alternative strategies for care, the reduction in admissions from the ED may also represent novel access to outpatient management strategies. Observation status may play a role in this, although evidence is mixed. While hospitals may be substituting observation stays for admissions for some patients,^37^ studies suggest only a slight increase in observation utilization before and after the HRRP after controlling for trends in targeted and nontargeted conditions.^3,13^ The sensitivity analyses in our study suggest that our results are not driven by observation stays, including our findings regarding CHF.

Earlier work has explored the potential for unintended harms to patients resulting from the HRRP, with mixed findings, reflecting different study designs and model specifications.^38,39,40,41,42^ A 2018 study by Wadhera et al^39^ observed an increase in 30-day mortality, which was likely driven by patients not readmitted within 30 days of discharge. Our study found that the HRRP was associated with a 1.2% decrease in the likelihood of readmission when recently discharged patients presented to the ED with conditions for which admission is potentially nondiscretionary, particularly CHF. This may represent a diversion into an alternative out-of-hospital pathway (eg, home nursing, urgent outpatient appointment).^43^ For example, case managers and utilization managers^44^ may suggest out-of-hospital management as a strategy to reduce HRRP penalties for patients presenting with CHF if they had a recent hospitalization. Such pathways may be intended to increase patient-centered care, but recent research suggests potential for increased morbidity and mortality.^45,46^ For instance, a 2017 study by Sax et al^45^ found that 1 in 3 patients with CHF discharged from the ED had a repeat ED visit, hospitalization, or death within 30 days after ED discharge. More research should examine this possibility.

This study suggests the need to better understand and account for the involvement of the ED in quality measurement for care coordination and population health management strategies within communities and health systems,^47^ particularly those involving hospital readmissions. Policy makers may wish to adopt proposals^48,49^ to incorporate ED utilization and observation stays into the HRRP to improve its reliability by better accounting for statistical outliers and reducing possible unintended consequences. Another possibility is to develop an explicit ED revisit measure that assesses readmissions, mortality, and morbidity after ED revisits.^50^ This would be justified by their prominence, as ED revisits account for nearly 40% of posthospitalization acute care encounters,^51^ and by their potential role in reducing readmissions.

### Limitations

Our study has some limitations. First, our study might have underestimated the reduction in readmissions from the ED after HRRP implementation. This might occur because, due to data restrictions, we included all Medicare patients instead of only fee-for-service Medicare patients who are included in readmission calculations for HRRP penalties. Additionally, our results may have been underestimated if hospitals anticipated the HRRP before implementation and thus changed their ED admitting behavior prior to that date or if there were spillover effects to nontargeted conditions.^3,13,22^

Second, we are limited by our data. Although we controlled for patient comorbidities, there might be unobserved differences among patients making ED revisits over time. In addition, coding of sepsis may have changed during the study.^52^ The difference-in-difference design makes these less likely to bias our results toward finding an effect, as there is no indication that patients with targeted (vs nontargeted) conditions are less sick after the HRRP, and if anything, patients revisiting the ED after HRRP implementatio may be more sick, as discharged patients are less likely to revisit the ED.

Third, our results may not be generalizable, as our analysis only includes 3 states. However, these states comprise approximately one-fourth of the country’s Medicare patients and one-fifth of HRRP-participating hospitals.

## Conclusion

Our findings suggest that the HRRP was associated with a decrease in the likelihood that hospitals would readmit recently discharged patients returning to the ED within 30 days of discharge. The decrease in ED readmissions was observed for patients diagnosed at the ED revisit with septicemia, AMI, acute cerebrovascular disease, and CHF—conditions for which admission is potentially nondiscretionary^15^—and particularly among patients presenting at the ED with CHF. Our findings provide insight into mechanisms underlying the changes in national readmission rates associated with implementation of the HRRP and raise concerns about potential unintended consequences of the program.

## Notes

Supplement. **eFigure 1.** Flowchart of Sample **eTable 1.** Unadjusted Index Hospitalizations Before or After Hospital Readmissions Reduction Program Implementation **eTable 2.** Whether an Initial Hospitalization Resulted in an Emergency Department Revisit **eAppendix 1.** Parallel Slopes Assumption **eFigure 2.** Unadjusted Trends in Admissions Before Hospital Readmissions Reduction Program Implementation **eAppendix 2.** Model **eAppendix 3.** Robustness Checks **eTable 3.** Whether an Emergency Department Revisit Resulted in a Readmission **eTable 4.** Whether an Emergency Department Revisit Resulted in a Readmission for Conditions for Which Admission Is Potentially Nondiscretionary **eTable 5.** Whether an Emergency Department Revisit Resulted in a Readmission, for Conditions for Which Admission Is Potentially Nondiscretionary Stratified by Condition **eTable 6.** Whether an Emergency Department Revisit Resulted in a Readmission for Conditions for Which Admission Is More Variable **eReferences.**
