# Supplementary material for: Assessment of Hospital Readmissions From the Emergency Department After Implementation of Medicare’s Hospital Readmissions Reduction Program
Source: JAMA Netw Open. 2020 May 1;3(5):e203857. doi: 10.1001/jamanetworkopen.2020.3857 (PMC7195622; doi:10.1001/jamanetworkopen.2020.3857)
Supplement: Supplement. — eFigure 1. Flowchart of Sample eTable 1. Unadjusted Index Hospitalizations Before or After Hospital Readmissions Reduction Program Implementation eTable 2. Whether an Initial Hospitalization Resulted in an Emergency Department Revisit eAppendix 1. Parallel Slopes Assumption eFigure 2. Unadjusted Trends in Admissions Before Hospital Readmissions Reduction Program Implementation eAppendix 2. Model eAppendix 3. Robustness Checks eTable 3. Whether an Emergency Department Revisit Resulted in a Readmission eTable 4. Whether an Emergency Department Revisit Resulted in a Readmission for Conditions for Which Admission Is Potentially Nondiscretionary eTable 5. Whether an Emergency Department Revisit Resulted in a Readmission, for Conditions for Which Admission Is Potentially Nondiscretionary Stratified by Condition eTable 6. Whether an Emergency Department Revisit Resulted in a Readmission for Conditions for Which Admission Is More Variable eReferences. [file jamanetwopen-3-e203857-s001.pdf]

Supplementary Online Content

Hsuan C, Carr BG, Hsia RY, Hoffman GJ. Assessment of hospital readmissions from the emergency department after implementation of Medicare's Hospital Readmissions Reduction Program. *JAMA Netw Open*. 2020;3(5):e203857. doi:10.1001/jamanetworkopen.2020.3857

eFigure 1. Flowchart of Sample
eTable 1. Unadjusted Index Hospitalizations Before or After Hospital Readmissions Reduction Program Implementation
eTable 2. Whether an Initial Hospitalization Resulted in an Emergency Department Revisit
eAppendix 1. Parallel Slopes Assumption
eFigure 2. Unadjusted Trends in Admissions Before Hospital Readmissions Reduction Program Implementation
eAppendix 2. Model
eAppendix 3. Robustness Checks
eTable 3. Whether an Emergency Department Revisit Resulted in a Readmission
eTable 4. Whether an Emergency Department Revisit Resulted in a Readmission for Conditions for Which Admission Is Potentially Nondiscretionary
eTable 5. Whether an Emergency Department Revisit Resulted in a Readmission, for Conditions for Which Admission Is Potentially Nondiscretionary Stratified by Condition
eTable 6. Whether an Emergency Department Revisit Resulted in a Readmission for Conditions for Which Admission Is More Variable
eReferences.

This supplementary material has been provided by the authors to give readers additional information about their work.

© 2020 Hsuan C et al. *JAMA Network Open.*

eFigure 1. Flowchart of Sample

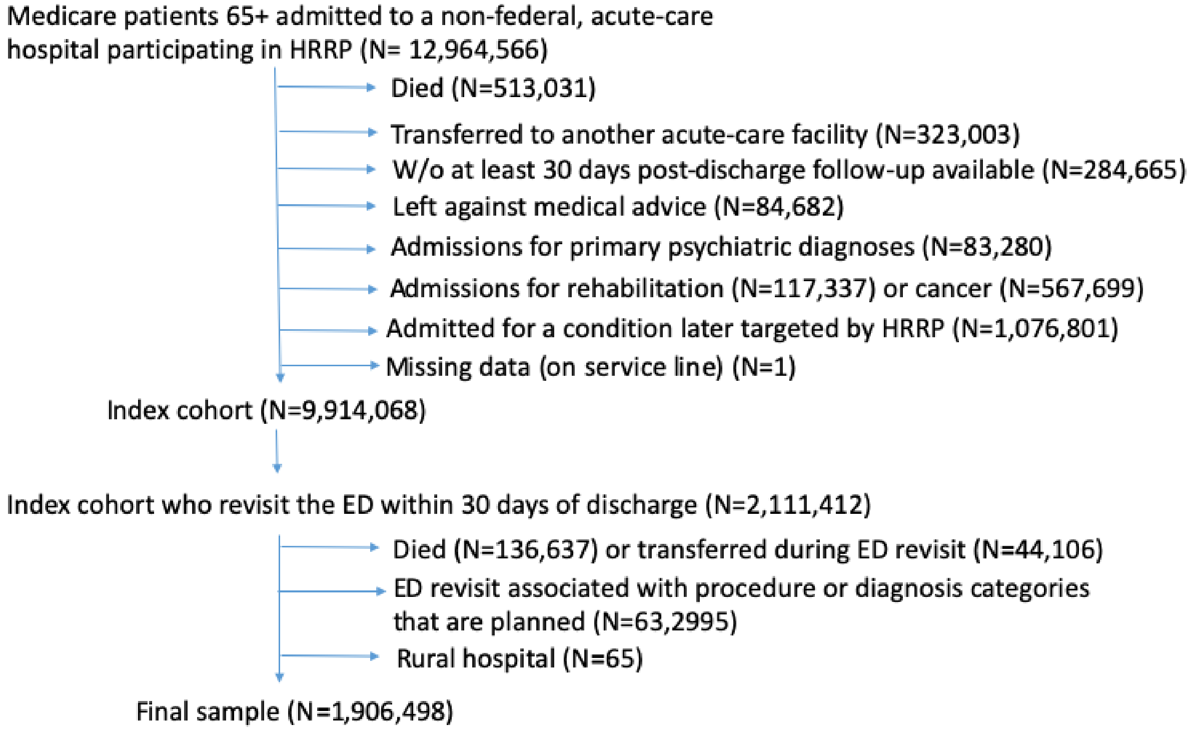

© 2020 Hsuan C et al. *JAMA Network Open.*

eTable 1. Unadjusted Index Hospitalizations Before or After Hospital Readmissions Reduction Program Implementation

	Total		Pre-HRRP implementation		Post-HRRP implementation	
	HRRP-targeted condition	Non-HRRP targeted condition	HRRP-targeted condition	Non-HRRP targeted condition	HRRP-targeted condition	Non-HRRP targeted condition
# of index hospitalizations	1,087,679	8,274,773	650,734	4,861,481	436,945	3,413,292
ED Revisits (both admit-and-release and admitted)						
Total	290,553 (26.7%)	1,820,804 (22.0%)	175,767 (27.0%)	1,065,035 (21.9%)	114,786 (26.3%)	755,769 (22.1%)
Unplanned	283,624 (26.4%)	1,773,286 (21.7%)	171,551 (26.7%)	1,036,487 (21.6%)	112,073 (26.0%)	736,799 (21.9%)
Readmissions of index hospitalizations						
Total	223,768 (20.6%)	1,318,543 (15.9%)	138,678 (21.3%)	792,853 (16.3%)	85,090 (19.5%)	525,690 (15.4%)
Unplanned	210,034 (19.6%)	1,211,373 (14.8%)	130,183 (20.3%)	726,391 (15.2%)	79,851 (18.5%)	484,982 (14.4%)
Of readmissions, from the ED						
Total	199,682 (89.2%)	1,118,863 (84.9%)	123,030 (88.7%)	665,807 (84.0%)	76,652 (90.1%)	453,056 (86.2%)
Unplanned	192,988 (91.9%)	1,073,119 (88.6%)	118,953 (91.4%)	638,252 (87.9%)	74,035 (92.7%)	434,867 (89.7%)

Abbreviations: ED = Emergency department; HRRP = Hospital Readmissions Reduction Program

Notes. Pre-HRRP implementation is Q1 2010-Q3 2012; post-HRRP implementation is Q4 2012-Q4 2014. All differences between index hospitalizations for HRRP-targeted conditions and non-targeted conditions are statistically significant ($p<0.001$).

Source. Authors' analysis of data from the Patient Discharge Data and Emergency Department and Ambulatory Surgery Data, Office of Statewide Health and Planning (OSHPD) (California), and the State Emergency Department Databases and the State Inpatient Databases from the Healthcare Cost and Utilization Project (New York and Florida)

© 2020 Hsuan C et al. *JAMA Network Open.*

eTable 2. Whether an Initial Hospitalization Resulted in an Emergency Department Revisit

	**Pre-Implementation**	**Post-Implementation**	**Post-Pre Difference**	**Difference-in-difference**
Targeted condition	0.246 [0.245, 0.248]	0.240 [0.239, 0.242]	-0.006*** [-0.008, -0.004]	-0.008*** [-0.010, -0.006]
Non-targeted condition	0.222 [0.221, 0.223]	0.224 [0.223, 0.225]	0.002** [0.001, 0.004]	

*p<0.05, **p<0.01, ***p<0.001

Notes. Predicted probabilities and marginal effects calculated from a linear probability model with hospital fixed effects. The model controls for age, sex, race/ethnicity, Charlson comorbidity index, whether the ED revisit was on a weekend, patient's condition at the index hospitalization and year. A negative difference or difference-in-difference indicates that ED revisits decreased post-implementation.

Source. Authors' analysis of data from the Patient Discharge Data and Emergency Department and Ambulatory Surgery Data, Office of Statewide Health and Planning (OSHPD) (California), and the State Emergency Department Databases and the State Inpatient Databases from the Healthcare Cost and Utilization Project (New York and Florida)

© 2020 Hsuan C et al. *JAMA Network Open.*

eAppendix 1. Parallel Slopes Assumption

In order for difference-in-differences to work properly, we need to satisfy the parallel slopes assumption. Specifically, this states that during the baseline period, readmissions from the ED of recently-discharged patients should be similar for the two comparison groups (the "intervention" group, ie patients at the index hospitalization who have conditions targeted by HRRP, versus the "control" group, ie patients at the index hospitalization who have conditions that are not targeted by HRRP).

Below are the unadjusted trends in admission before HRRP implementation. These suggest that the parallel slopes assumption was met.

In addition, we empirically tested the parallel slopes assumption by narrowing the sample to the pre-period (ie limiting the sample to before HRRP implementation) and falsely creating a "post" indicator for January 1, 2011. The pre-period placebo test yielded a difference-in-difference estimate of -0.001 (95% CI: -0.007, 0.004, p=0.621), which suggests that the groups did not change in trends before and after the false post indicator of January 1, 2011.

© 2020 Hsuan C et al. *JAMA Network Open.*

eFigure 2. Unadjusted Trends in Admissions Before Hospital Readmissions Reduction Program Implementation

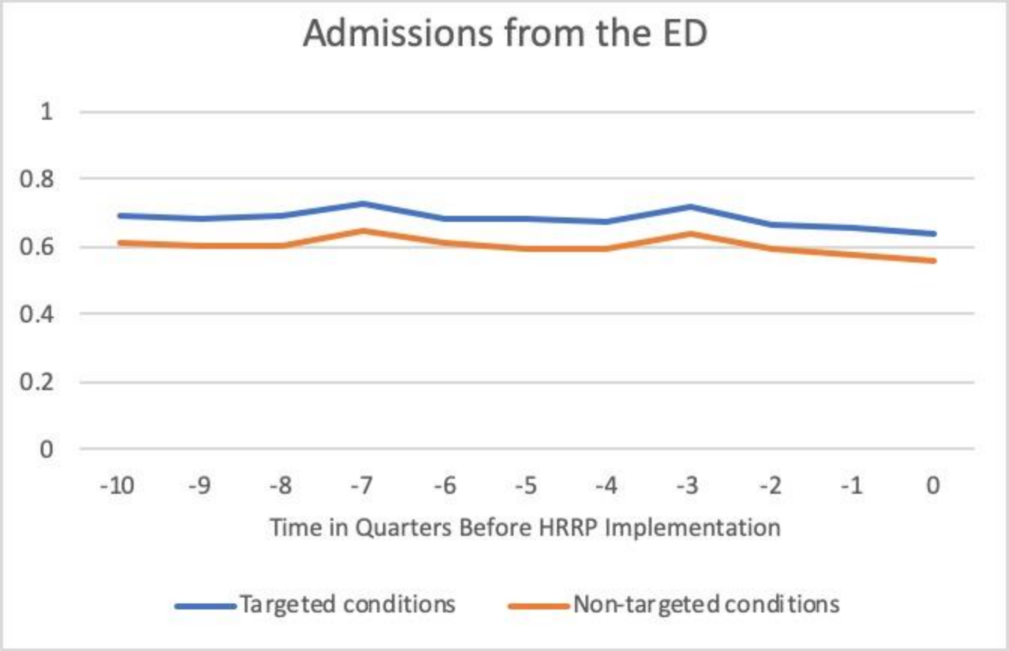

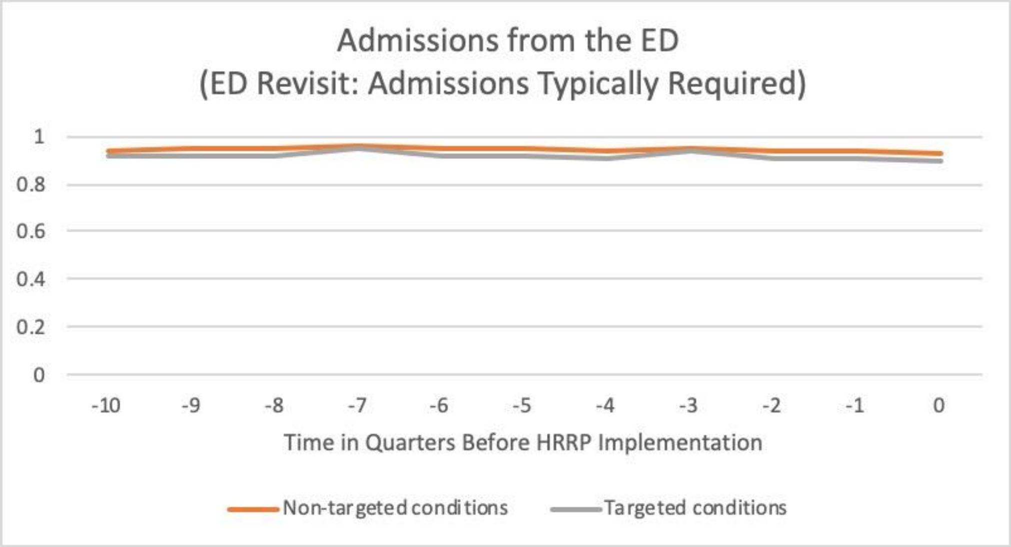

© 2020 Hsuan C et al. *JAMA Network Open.*

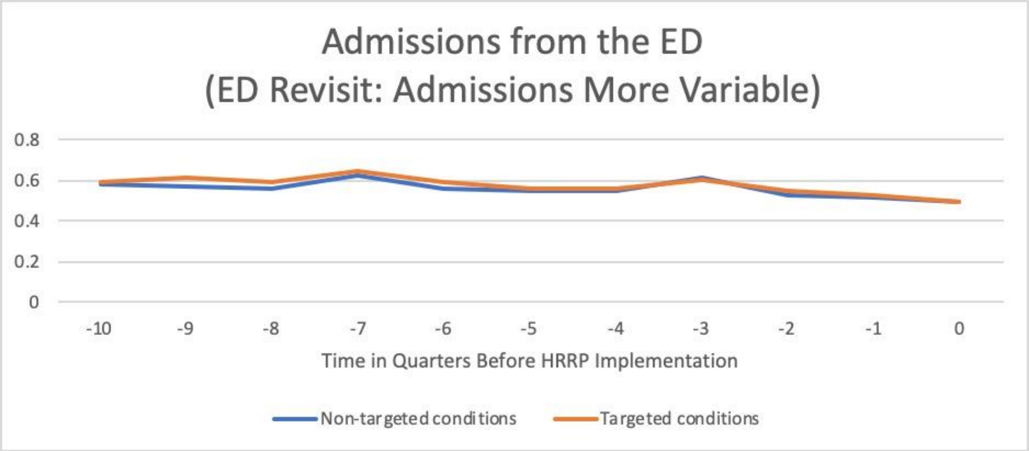

Source. Authors' analysis of data from the Patient Discharge Data and Emergency Department and Ambulatory Surgery Data, Office of Statewide Health and Planning (OSHPD) (California), and the State Emergency Department Databases and the State Inpatient Databases from the Healthcare Cost and Utilization Project (New York and Florida)

© 2020 Hsuan C et al. *JAMA Network Open.*

eAppendix 2. Model

For discharge *i* in hospital h, we used linear regression with hospital clustered standard errors to model:

$$Y_{ih} = \alpha + \beta_1 Post_{ih} + \beta_2\, Target_{ih} + \beta_3 Post_{ih} \text{ x } Target_{ih} + \beta_4 Risk_{ih} + \beta_5\, Hosp_h + \beta_6\, State_h + \beta_7\, Year,$$

where Y is whether the patient was readmitted during the ED revisit; *Post* is whether the ED revisit was after HRRP implementation; *Target* is whether the patient's initial hospitalization was for a condition targeted by HRRP; *Risk* are patient risk adjustment factors, including: age, sex, race, Charlson comorbidity index, the readmission cohort (as defined by CMS), and whether the ED revisit was on a weekend; *Hosp* are hospital characteristics, including academic status, size, urbanicity, and ownership; *State* is the state in which the hospital is located; and *Year* is the year the discharge patient revisited the ED. Age was by 5-year bands until age 85; all patients 85 years or older were grouped together. Academic status was either an accredited program in the Accreditation Council for Graduate Medical Education or Member of Council of Teaching Hospital of the Association of American Medical Colleges. Hospital size was based on quintiles of admission. Metropolitan or urban designation was based on the Rural-Urban Continuum Codes, which we further classified into whether the hospital was in a metropolitan or urban non-metropolitan area (as described in the paper, we excluded rural hospitals).

© 2020 Hsuan C et al. *JAMA Network Open.*

eAppendix 3. Robustness Checks

We conducted several robustness checks. First, we examined alternative model specifications (logistic model and hospital fixed effects). Second, we modified the outcome to account for potential differences in care patterns. Specifically, we defined the outcome variable to be admission or transfer, or admission or observation stay. Observation stay information was available only for two of the three states in our study (Florida and New York). Third, previous research[1-3] suggests that community factors, including availability of post-acute care, influence readmissions. Following previous research, we included additional covariates in our model for population size, unemployment, median household income, percent poverty, percent uninsured, percent of the population in deep poverty, population in nursing homes, percent non-English speaking population, percent vacant housing, educational attainment, number of nursing facilities per 100K, primary care providers per 100K, number of specialists per 100K, number of cardiologists per 100K, number of community health centers per 100K, the ratio of primary care physicians to specialists, the Medicare population per 100K, the number of federally-qualified health centers per 100K, and the number of hospital beds per 100K. Fourth, in the main analysis, we adjusted for changes in electronic transmission standards that increased the number of diagnosis codes and that may make patients appear more sick.[4] We did this by adjusting our Charlson index so that they use only the first 9 secondary diagnosis codes. In the robustness check, we used all secondary diagnosis codes in calculating the Charlson index. Fifth, we added dummy variables for time (represented by separate variables for each month of the study period, except the first) to account for overall time trends. Note that we do not include this in the main analysis because only two states provided month. Sixth, we examined our exclusion of conditions later targeted by HRRP. Seventh, we conducted a falsification test where we examine readmission patterns for patients revisiting the ED 31 to 45 days after admission. Eighth, for conditions at the ED where admission is more variable, we exclude mood disorders.

The results from these robustness checks are below.

© 2020 Hsuan C et al. *JAMA Network Open.*

eTable 3. Whether an Emergency Department Revisit Resulted in a Readmission

a. Linear probability model

	Main Analysis	Including Transfers	Community factors	Hospital FE	31-45 days	Observation status	Comorbidities using all secondary diagnosis codes	Monthly time trends	Later targeted conditions	Including patients who die at the revisit
Post	-0.001 [-0.006, 0.005]	-0.001 [-0.006, 0.004]	0.002 [-0.004, 0.007]	-0.001 [-0.005, 0.003]	-0.003 [-0.012, 0.007]	0.001 [-0.005, 0.006]	0.000 [-0.005, 0.006]	0.010* [0.002, 0.019]	-0.001 [-0.006, 0.005]	-0.002 [-0.008, 0.003]
Applicable condition	0.064*** [0.060, 0.068]	0.064*** [0.060, 0.068]	0.064*** [0.059, 0.068]	0.062*** [0.058, 0.065]	0.057*** [0.050, 0.065]	0.064*** [0.060, 0.068]	0.061*** [0.057, 0.065]	0.060*** [0.055,0.065]	0.000 [0.004,0.006]	0.072*** [0.067, 0.076]
Post x Applicable Condition	-0.009*** [-0.013, -0.004]	-0.009*** [-0.013, -0.004]	-0.009*** [-0.013, -0.004]	-0.008*** [-0.012, -0.004]	-0.007 [-0.016, 0.001]	-0.001*** [-0.005, -0.004]	-0.009*** [-0.014, -0.005]	-0.009** [-0.015, -0.003]	-0.012*** [-0.017, -0.007]	-0.007* [-0.011, -0.002]
N	1,906,498	1,942,786	1,906,498	1,906,498	432,504	1,906,498	1,906,498	1,236,545	1,841,233	2,020,615
R2	0.032	0.030	0.0369	0.0144	0.0315	0.0332	0.0364	0.0335	0.0327	0.0147
R2 within				0.0174						
R2 between				0.0032						

*p<0.05, **p<0.01, ***p<0.001

Notes. Linear probability model with robust clustered standard errors for the hospital. The model controls for age, sex, race/ethnicity, Charlson comorbidity index, whether the ED revisit was on a weekend, patient's condition at the index hospitalization, hospital size, teaching status, metropolitan or urban designation, ownership status, state, and year. In addition, each of the sensitivity analyses includes the variables as described in the text

© 2020 Hsuan C et al. *JAMA Network Open.*

b. Logistic regression

	Odds ratio
Post	0.998<br>[0.974, 1.020]
Applicable condition	1.331***<br>[1.306, 1.356]
Post x Applicable Condition	0.952***<br>[0.933, 0.972]
N	1,906,498
Pseudo-R2	0.0241

Notes. Logistic regression with robust clustered standard errors for the hospital. The model controls for age, sex, race/ethnicity, Charlson comorbidity index, whether the ED revisit was on a weekend, patient's condition at the index hospitalization, hospital size, teaching status, metropolitan or urban designation, ownership status, state, and year. A difference-in-difference less than one indicates that readmissions from the ED decreased post-implementation for recently-discharged patients.

Source. Authors' analysis of data from the Patient Discharge Data and Emergency Department and Ambulatory Surgery Data, Office of Statewide Health and Planning (OSHPD) (California), and the State Emergency Department Databases and the State Inpatient Databases from the Healthcare Cost and Utilization Project (New York and Florida)

© 2020 Hsuan C et al. *JAMA Network Open.*

eTable 4. Whether an Emergency Department Revisit Resulted in a Readmission for Conditions for Which Admission Is Potentially Nondiscretionary[a]

a. Linear probability model for all conditions where admission is potentially non-discretionary

	Main Analysis	Including Transfers	Community factors	Hospital FE	31-45 days	Observation status	Comorbidities using all secondary diagnosis codes	Monthly time trends	Later targeted conditions	Including patients who die at the revisit
Post	0.002 [-0.003, 0.007]	0.002 [-0.003, 0.007]	0.003 [-0.002, 0.008]	0.002 [-0.003, 0.007]	0.007 [-0.001, 0.016]	0.003 [-0.002, 0.008]	0.003 [-0.002, 0.008]	0.006 [-0.002, 0.015]	0.002 [-0.003, 0.007]	0.001 [-0.004, 0.006]
Applicable condition	0.001 [-0.004, 0.005]	0.001 [-0.003, 0.006]	0.001 [-0.003, 0.006]	0.000 [-0.004, 0.005]	0.004 [-0.004, 0.012]	0.000 [-0.004, 0.005]	0.000 [-0.004, 0.005]	-0.001 [-0.006, 0.005]	0.014*** [-0.008, 0.020]	-0.000 [-0.004, 0.004]
Post x Applicable Condition	-0.010*** [-0.015, -0.004]	-0.011*** [-0.016, -0.006]	-0.011*** [-0.016, -0.006]	-0.010*** [-0.016, -0.005]	-0.008 [-0.018, 0.002]	-0.010*** [-0.015, -0.004]	-0.010*** [-0.015, -0.005]	-0.012** [-0.019, -0.004]	0.000 [-0.006, 0.008]	-0.010*** [-0.015, -0.005]
N	280,575	290,608	280,575	280,575	64,310	280,575	280,575	188,201	232,432	329,250
R2	0.0248	0.0231	0.0289	0.0081	0.0213	0.0260	0.0257	0.0273	0.0214	0.0136
R2 within				0.0082						
R2 between				0.0606						

*p<0.05, **p<0.01, ***p<0.001

Notes. Linear probability model with robust clustered standard errors for the hospital. The model controls for age, sex, race/ethnicity, Charlson comorbidity index, whether the ED revisit was on a weekend, patient's condition at the index hospitalization, hospital size, teaching status, metropolitan or urban designation, ownership status, state, and year. In addition, each of the sensitivity analyses includes the variables as described above. A negative difference-in-difference indicates that readmissions from the ED decreased post-implementation for recently-discharged patients.
[a] ED diagnoses where admission is potentially non-discretionary are: septicemia, AMI, acute cerebrovascular disease, and congestive heart failure.[5]

Source. Authors' analysis of data from the Patient Discharge Data and Emergency Department and Ambulatory Surgery Data, Office of Statewide Health and Planning (OSHPD) (California), and the State Emergency Department Databases and the State Inpatient Databases from the Healthcare Cost and Utilization Project (New York and Florida)

© 2020 Hsuan C et al. *JAMA Network Open.*

b. Logistic regression

	Odds ratio
Post	1.04<br>[0.92, 1.16]
Applicable condition	0.99<br>[0.93, 1.06]
Post x Applicable Condition	0.90**<br>[0.84, 0.97]
N	280,575
Pseudo-R2	0.0529

*p<0.05, **p<0.01, ***p<0.001

Notes. Logistic regression with robust clustered standard errors for the hospital. The model controls for age, sex, race/ethnicity, Charlson comorbidity index, whether the ED revisit was on a weekend, patient's condition at the index hospitalization, hospital size, teaching status, metropolitan or urban designation, ownership status, state, and year. A difference-in-difference less than one indicates that readmissions from the ED decreased post-implementation for recently-discharged patients.

Source. Authors' analysis of data from the Patient Discharge Data and Emergency Department and Ambulatory Surgery Data, Office of Statewide Health and Planning (OSHPD) (California), and the State Emergency Department Databases and the State Inpatient Databases from the Healthcare Cost and Utilization Project (New York and Florida)

© 2020 Hsuan C et al. *JAMA Network Open.*

eTable 5. Whether an Emergency Department Revisit Resulted in a Readmission, for Conditions for Which Admission Is Potentially Nondiscretionary Stratified by Condition[a]

a. Presenting in ED with CHF

i. Linear Probability Model

	Main Analysis	Including Transfers	Community factors	Hospital FE	31-45 days	Observation status	Comorbidities using all secondary diagnosis codes	Monthly time trends	Later targeted conditions	Including patients who die at the revisit
Post	0.002 [-0.008, 0.013]	0.002 [-0.008, 0.012]	0.003 [-0.007, 0.014]	0.002 [-0.008, 0.013]	0.010 [-0.007, 0.28]	0.004 [-0.006, 0.014]	0.002 [-0.008, 0.012]	0.005 [-0.013, 0.023]	0.002 [-0.009, 0.013]	0.002 [-0.008, 0.012]
Applicable condition	0.011*** [0.005, 0.018]	0.011*** [0.005, 0.017]	0.011** [0.004, 0.017]	0.009** [0.003, 0.014]	0.008 [-0.003, 0.020]	0.011*** [0.005, 0.017]	0.012*** [0.005, 0.018]	0.011* [0.003, 0.020]	0.012* [0.002, 0.021]	0.011** [0.005, 0.017]
Post x Applicable Condition	-0.012** [-0.020, -0.004]	-0.011** [-0.019, -0.004]	-0.011** [-0.019, -0.003]	-0.009** [-0.017, -0.002]	-0.002 [-0.017, 0.014]	-0.012** [-0.020, -0.005]	-0.012** [-0.020, -0.004]	-0.010 [-0.022, 0.001]	-0.001 [-0.013, 0.010]	-0.011** [-0.018, -0.004]
N	123,981	127,017	123,981	123,981	28,621	123,981	123,981	78,101	91,139	130,854
R2	0.037	0.0353	0.044	0.0021	0.0373	0.0403	0.0372	0.0352	0.0344	0.0348
R2 within				0.0037						
R2 between				0.0002						

*p<0.05, **p<0.01, ***p<0.001

© 2020 Hsuan C et al. *JAMA Network Open.*

ii. Logistic regression

	Odds ratio
Post	1.007 [-.883, 1.148]
Applicable condition	1.123** [1.051, 1.200]
Post x Applicable Condition	0.895** [0.826, 0.971]
N	123,871
Pseudo-R2	0.0506

*p<0.05, **p<0.01, ***p<0.001

b. Presenting with septicemia, AMI, acute cerebrovascular disease

	Main Analysis	Including Transfers	Community factors	Hospital FE	31-45 days	Observati on status	Comorbidities using all secondary diagnosis codes	Monthly time trends	Later targeted conditions	Including patients who die at the revisit
Post	0.002 [-0.003, 0.006]	0.001 [-0.003, 0.006]	0.002 [-0.002, 0.007]	0.001 [-0.004, 0.007]	0.001 [-0.007, 0.010]	0.002 [-0.003, 0.006]	0.001 [-0.003, 0.006]	0.007 [-0.000, 0.013]	0.000 [-0.004, 0.004]	0.001 [-0.003, 0.004]
Applicable condition	-0.008*** [-0.013, -0.004]	-0.007*** [-0.011, -0.003]	-0.008*** [-0.012, -0.004]	-0.009*** [-0.013, -0.004]	0.003 [-0.005, 0.011]	-0.008** [-0.012, -0.003]	-0.008*** [-0.013, -0.004]	-0.009** [-0.015, -0.004]	0.003 [-0.002, 0.007]	-0.008*** [-0.011, -0.004]
Post x Applicable Condition	-0.001 [-0.007, 0.004]	-0.001 [-0.006, 0.003]	-0.002 [-0.007, 0.004]	-0.001 [-0.006, 0.004]	-0.009 [-0.018, 0.000]	-0.001 [-0.006, 0.004]	-0.001 [-0.007, 0.004]	-0.002 [-0.009, 0.005]	0.002 [-0.004, 0.008]	-0.000 [-0.005, 0.004]
N	156,594	163,591	156,594	156,594	35,689	156, 594	156,594	110,100	184,505	198,396

© 2020 Hsuan C et al. *JAMA Network Open.*

R2	0.0222			0.0142	0.0104	0.0222	0.0223	0.0240	0.0163	0.0161
R2 within				0.0140						
R2 between				0.0351						

*p<0.05, **p<0.01, ***p<0.001

i. Logistic regression

	Odds ratio
Post	1.082<br>[0.873, 1.342]
Applicable condition	0.735***<br>[0.623, 0.867]
Post x Applicable Condition	0.962<br>[0.803, 1.152]
N	156,594
Pseudo-R2	0.0675

*p<0.05, **p<0.01, ***p<0.001

Notes. Linear probability model or logistic regression model with robust clustered standard errors for the hospital. The model controls for age, sex, race/ethnicity, Charlson comorbidity index, whether the ED revisit was on a weekend, patient's condition at the index hospitalization, hospital size, teaching status, metropolitan or urban designation, ownership status, state, and year. In addition, each of the sensitivity analyses includes the variables as described above. A negative difference-in-difference indicates that readmissions from the ED decreased post-implementation for recently-discharged patients.
[a] ED diagnoses where admission is potentially non-discretionary are: septicemia, AMI, acute cerebrovascular disease, and congestive heart failure.[5],

© 2020 Hsuan C et al. *JAMA Network Open.*

eTable 6. Whether an Emergency Department Revisit Resulted in a Readmission for Conditions for Which Admission Is More Variable[a]

a. Linear probability model

	Main Analysis	Including Transfers	Community factors	Hospital FE	31-45 days	Observation status	Comorbidities using all secondary diagnosis codes	Monthly time trends	Later targeted conditions	Including patients who die at the revisit	Excluding pts presenting with mood disorders
Post	0.011 [-0.004, 0.024]	0.009 [-0.005, 0.023]	0.014* [0.000, 0.024]	0.008 [-0.005, 0.022]	-0.004 [-0.027, 0.019]	0.011 [-0.003, 0.025]	0.011 [-0.003, 0.025]	0.014 [-0.008, 0.036]	0.004 [-0.009, 0.017]	0.009 [-0.005, 0.023]	0.009 [-0.005, 0.023]
Applicable condition	0.020*** [0.009, 0.031]	0.021*** [0.001, 0.033]	0.023*** [0.012, 0.034]	0.024*** [0.014, 0.035]	0.009 [-0.013, 0.032]	0.022*** [0.011, 0.033]	0.021** [0.010, 0.032]	0.013 [-0.000, 0.027]	0.068 [0.058, 0.079]	0.025*** [0.014, 0.037]	0.020*** [0.009, 0.032]
Post x Applicable Condition	-0.008 [-0.021, 0.004]	-0.008 [-0.021, 0.004]	-0.010 [-0.022, 0.003]	-0.006 [-0.019, 0.006]	0.003 [-0.024, 0.029]	-0.005 [-0.018, 0.008]	-0.005 [-0.018, 0.007]	-0.007 [-0.023, 0.010]	-0.007 [-0.018, 0.004]	-0.009 [-0.021, 0.004]	-0.009 [-0.022, 0.004]
N	194,344	196,629	194,344	194,344	50,878	194,344	194,344	122,402	212,951	196,479	190,194
R2	0.0551	0.0532	0.0690	0.0178	0.0544	0.0601	0.0610	0.0593	0.0647	0.0384	0.0550
R2 within				0.0242							
R2 between				0.0098							

*p<0.05, **p<0.01, ***p<0.001

Notes. Linear probability model with robust clustered standard errors for the hospital. The model controls for age, sex, race/ethnicity, Charlson comorbidity index, whether the ED revisit was on a weekend, patient's condition at the index hospitalization, hospital size, teaching status, metropolitan or urban designation, ownership status, state, and year. In addition, each of the sensitivity analyses includes the variables as described above. A negative difference-in-difference indicates that readmissions from the ED decreased post-implementation for recently-discharged patients.
[a] Discretionary ED diagnoses are five ED diagnoses for which patient admissions to the hospital are more variable: mood disorders, nonspecific chest pain, skin and soft tissue infections, urinary tract infections, and COPD.[5]

Source. Authors' analysis of data from the Patient Discharge Data and Emergency Department and Ambulatory Surgery Data, Office of Statewide Health and Planning (OSHPD) (California), and the State Emergency Department Databases and the State Inpatient Databases from the Healthcare Cost and Utilization Project (New York and Florida)

© 2020 Hsuan C et al. *JAMA Network Open.*

b. Logistic regression

	Odds ratio
Post	1.05<br>[0.98, 1.11]
Applicable condition	1.10***<br>[1.05, 1.15]
Post x Applicable Condition	0.98<br>[0.92, 1.03]
N	194,344
Pseudo-R2	0.0446

*p<0.05, **p<0.01, ***p<0.001

Notes. Logistic regression with robust clustered standard errors for the hospital. The model controls for age, sex, race/ethnicity, Charlson comorbidity index, whether the ED revisit was on a weekend, patient's condition at the index hospitalization, hospital size, teaching status, metropolitan or urban designation, ownership status, state, and year. A difference-in-difference less than one indicates that readmissions from the ED decreased post-implementation for recently-discharged patients.

[a] These are five ED diagnoses for which patient admissions to the hospital are highly variable: mood disorders, nonspecific chest pain, skin and soft tissue infections, urinary tract infections, and COPD.[5]

Source. Authors' analysis of data from the Patient Discharge Data and Emergency Department and Ambulatory Surgery Data, Office of Statewide Health and Planning (OSHPD) (California), and the State Emergency Department Databases and the State Inpatient Databases from the Healthcare Cost and Utilization Project (New York and Florida)

© 2020 Hsuan C et al. *JAMA Network Open.*

eReferences.

1. Herrin J, St. Andre J, Kenward K, Joshi MS, Audet AMJ, Hines SC. Community factors and hospital readmission rates. *Health services research.* 2015;50(1):20-39.
2. Gaskin DJ, Zare H, Vazin R, Love D, Steinwachs D. Racial and Ethnic Composition of Hospitals' Service Areas and the Likelihood of Being Penalized for Excess Readmissions by the Medicare Program. *Medical care.* 2018;56(11):934-943.
3. Nagasako EM, Reidhead M, Waterman B, Dunagan WC. Adding socioeconomic data to hospital readmissions calculations may produce more useful results. *Health affairs (Project Hope).* 2014;33(5):786-791.
4. Ody C, Msall L, Dafny LS, Grabowski DC, Cutler DM. Decreases In Readmissions Credited To Medicare's Program To Reduce Hospital Readmissions Have Been Overstated. *Health affairs (Project Hope).* 2019;38(1):36-43.
5. Venkatesh AK, Dai Y, Ross JS, Schuur JD, Capp R, Krumholz HM. Variation in US hospital emergency department admission rates by clinical condition. *Medical care.* 2015;53(3):237-244.

© 2020 Hsuan C et al. *JAMA Network Open.*
